# Efficacy and safety of endoscopic “calabash” ligation and resection for small gastric stromal tumors originating from the muscularis propria

**DOI:** 10.1002/cam4.5471

**Published:** 2022-12-12

**Authors:** Min‐Si Peng, Hao‐Tian Zeng, Zhu‐Liang Zhang, Ze‐Ming Chen, Ting Long, Li‐Sheng Wang, Zheng‐Lei Xu

**Affiliations:** ^1^ The Second Clinical Medical College, Jinan University Shenzhen Guangdong Province China; ^2^ Department of Pathology The Second Clinical Medical College Jinan University (Shenzhen People's Hospital) Shenzhen Guangdong Province China; ^3^ Department of Gastroenterology The Second Clinical Medical College, Jinan University (Shenzhen People's Hospital) Shenzhen Guangdong Province China

**Keywords:** endophytic type, endoscopic “calabash” ligation and resection (ECLR), endoscopic submucosal excision (ESE), gastric stromal tumor (GST), muscularis propria

## Abstract

**Aim:**

We compared endoscopic “calabash” ligation and resection (ECLR) and endoscopic submucosal excision (ESE) in treating endophytic gastric stromal tumors (GSTs) ≤15 mm in diameter originating from the muscularis propria.

**Methods:**

We performed a retrospective study and included patients who visited our hospital for removal of small endophytic GSTs (diameter ≤ 15 mm) confirmed by postoperative pathological reports between February 2019 and December 2020. Patients were assigned to the study (received ECLR) or control (accepted ESE) groups, and their medical records were reviewed. Age, sex, GST size, resection outcomes, procedure measurements, lengths of hospital stays, medical expenses, intraoperative and postoperative complications, and follow‐up outcomes were documented and compared between the two groups. Propensity score matching was used to avoid retrospective biases.

**Results:**

A total of 277 patients were included in the analysis, with 135 in the study group and 142 in the control group. After propensity score matching, 119 cases in each group were finally included in the study. Compared to the control group, the study group had significantly shorter procedure durations and lengths of hospital stays, as well as reduced medical expenses. Compared to the control group, the study group also had significantly lower incidence rates of intraoperative stomach perforation, postoperative intraperitoneal infection, and postoperative electrocoagulation syndrome, as well as a lower intensity of postoperative pain. There were no significant differences in the other measurements between the two groups.

**Conclusion:**

ECLR is an effective and safe procedure for treating patients with endophytic GSTs ≤15 mm in diameter originating from the muscularis propria.

## Graphical abstract

Two groups of patients with small gastric stromal tumors were collected and received ECLR and ESE, respectively. The differences in operation time, operation effect, complications, length of hospital stay, and cost were compared between the two groups. The results showed that ECLR surgery was better than ESE surgery.

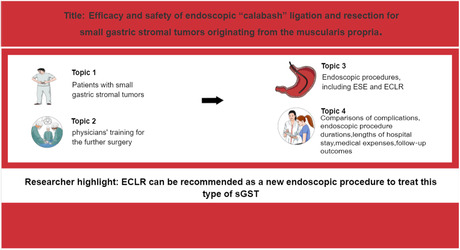

## 1 INTRODUCTION

Gastrointestinal stromal tumors originate from the interstitial cells of Cajal in the stomach and intestine and are the most common mesenchymal tumors of the gastrointestinal tract.^1^ The most common gastrointestinal stromal tumor is gastric stromal tumor (GST), accounting for 55.6% of all gastrointestinal stromal tumors.^2^ With the advances and broad application of the electronic endoscope (hereafter referred to as the endoscope), an increasing number of small GSTs (sGSTs, diameter <2 cm) with no obvious symptoms that increase the number of GSTs with clinical symptoms have been reported.^3^

Previous studies have shown that sGSTs have very low proliferative activity^4^, ^5^ and inert biological properties, especially small GSTs <1 cm in diameter. However, some other studies have shown that sGSTs carry a high risk of malignant transformation.^6^, ^7^, ^8^, ^9^ Patients with sGSTs were recommended to undergo invasive examinations, including endoscopy and endoscopic ultrasound (EUS), during follow‐up visits; however, long‐term and high‐frequency follow‐ups could lead to a greater economic burden on these patients. Meanwhile, those who did not participate in the follow‐up had greater psychological stress levels and a lower quality of life. Therefore, most patients with sGSTs want treatment as early as possible.^10^

Currently, endoscopic treatment of GSTs is highly effective, with outcomes similar to those attained by traditional or laparoscopic surgery in patients with GSTs <5 cm in diameter. Endoscopic treatment takes less time and causes minor trauma, with fewer complications, faster recovery, and lower costs.^11^, ^12^ ESE is currently recommended as a feasible treatment approach for sGSTs originating from the muscularis propria.^13^ Jeong et al. reported that ESE treatment could achieve a high complete resection rate with a low incidence of complications in patients with GSTs originating from the muscularis propria.^14^ However, this technique requires physicians to have significant technical skills and undergo extensive training. Here, we designed a simple surgical method for the treatment of sGSTs. Following adequate preliminary research, we obtained ethical approval and a new technology certification from the Shenzhen People's Hospital in December 2018. We have christened this method “endoscopic ‘calabash’ ligation and resection” (ECLR). In the present study, we focused on endophytic sGSTs ≤15 mm in diameter originating from the muscularis propria and studied the efficacy and safety of ECLR in this context.

## 2 MATERIALS AND METHODS

### 2.1 Study design, participant selection, and group assignments

We performed a retrospective study and analyzed patients with GST confirmed by endoscopic biopsy at our hospital between February 2019 and December 2020. The study protocol was approved by the hospital ethics committee.

The inclusion criteria were as follows: (a) ages between 16–80 years; (b) presence of endophytic GST originating from the gastric muscularis propria confirmed by preoperative EUS examination; (c) no evidence of lymph node or distant metastasis on preoperative CT or magnetic resonance imaging examinations; (d) GST ≤15 mm in diameter; and (e) treatment completed by either ESE or ECLR. The study exclusion criteria were as follows: (a) presence of severe comorbidities, such as heart, liver, kidney, or hematopoietic illness; (b) presence of other diseases that could significantly extend the procedure duration or the length of hospital stay; and (c) existence of incomplete medical records.

The study participants were assigned to either a control group (GST patients treated with ESE) or a study group (GST patients treated with ECLR).

### 2.2 Details of physicians' training to standardize their technical skills

All participating endoscopic doctors in Shenzhen People's Hospital were attending physicians associated with chief physicians or chief physicians, and all had adequate proficiency in ESE and ECLR procedures. Additionally, each surgeon independently completed >300 cases of four‐level digestive endoscopic surgery and received standardized training in the procedure steps and instruments before the clinical application of ESE and ECLR.

### 2.3 Operating instruments

**FIGURE 1:**
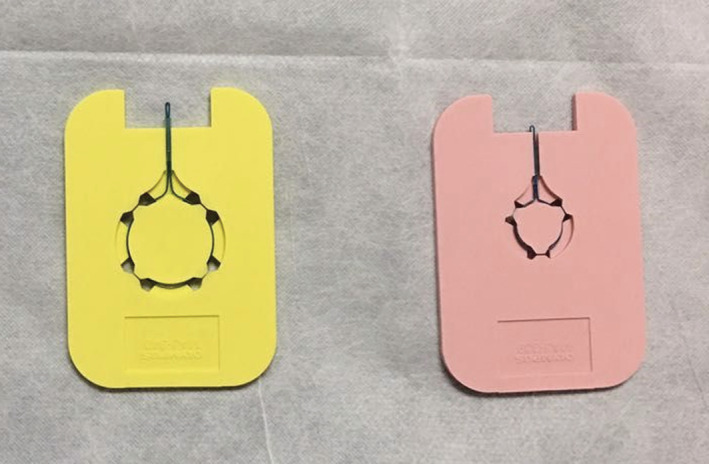
MAJ‐340 and MAJ‐339 nylon rings.

**FIGURE 2:**
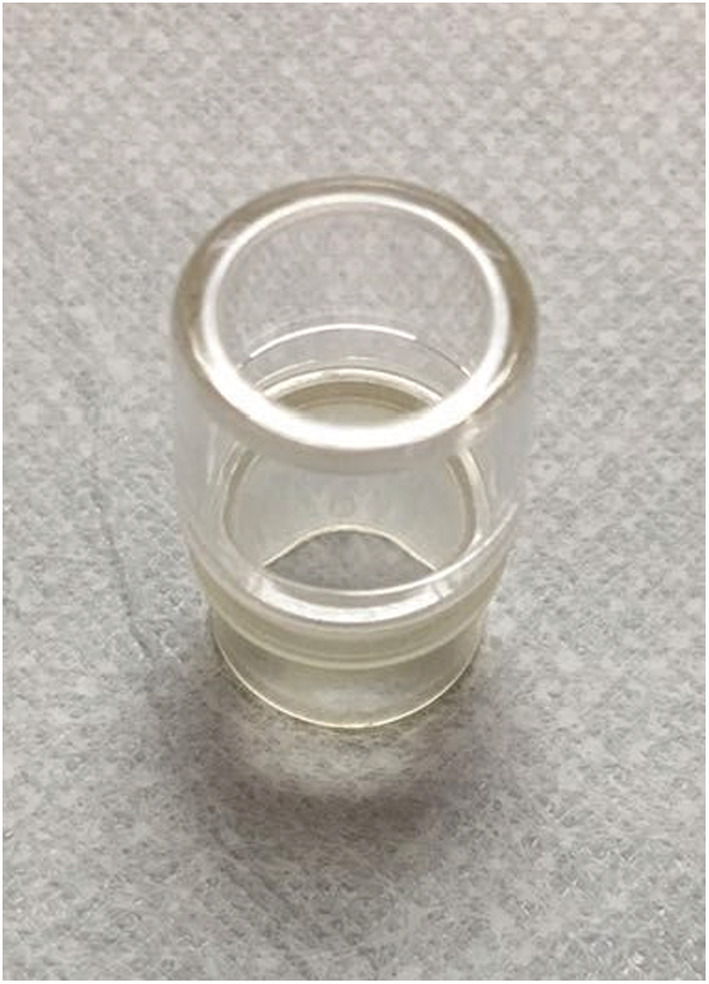
Transparent ligation cap.

The instruments used in this study included an electronic endoscope (GIF‐Q260J Gastroscope; Olympus Corporation, Tokyo, Japan), ultrasound endoscope (SU‐9000 Circular Scan Ultrasound Endoscope; Hitachi High‐Technologies Corporation, Tokyo, Japan), argon air knife (VIO‐200 S; ERBE Elektromedizin GmbH, Tübingen, Germany), IT‐Knife2 (KD‐611L; Olympus Corporation, Tokyo, Japan), DualKnife (KD‐650L; Olympus Corporation, Tokyo, Japan), disposable multi‐functional knife (Anrui, Zhejiang, China), disposable injection needle (NM‐200L‐0423; Olympus Corporation, Tokyo, Japan), disposable snare (MTNPFS01‐02423180; Nanwei Medical Technology Co., Ltd., Nanjing, China), hot biopsy forceps (HBF‐16/1800; Nanwei Medical Technology Co., Ltd., Nanjing, China), titanium clip (ROCC‐D‐26‐195; Nanwei Medical Technology Co., Ltd., Nanjing, China), transparent peeling cap (Olympus Corporation, Tokyo, Japan), nylon ligation ring (Figure 1) (Olympus Corporation, Tokyo, Japan), and transparent ligation cap (Figure 2) (Olympus Corporation, Tokyo, Japan). The fluid used for submucosal injection was composed of 250 ml saline, 0.5 mg methylene blue, and 1 mg adrenaline. Intravenous anesthesia was administered with midazolam, meperidine, or propofol.

### 2.4 Endoscopic procedures

#### 2.4.1 ESE with the circumferential incision

**FIGURE 3:**
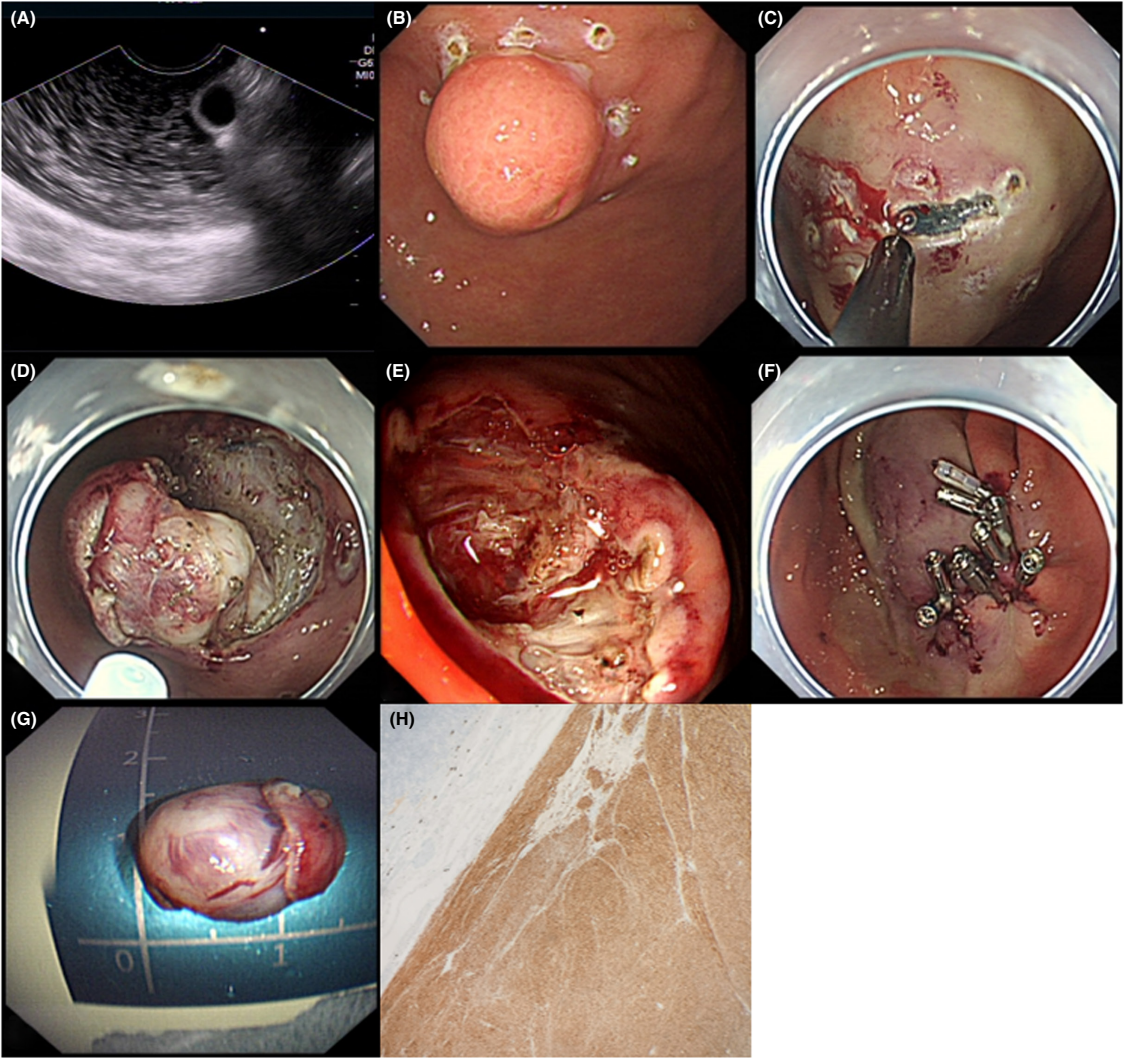
Endoscopic submucosal excavation circumferential incision approach. (A) endoscopic ultrasound examination showing that the tumor originated from the muscularis propria and was growing toward the gastric lumen. (B) Electrocoagulation was used to circumferentially mark the gastric stromal tumor (GST). (C) Submucosal injection followed by a circumferential incision of the mucosa was performed to open the mucosa and submucosa layers surrounding the gastric submucosal tumors (SMT). (D) The white tumor body was closely connected to the muscularis propria after incising the submucosa around the GST. Part of the muscularis propria was excavated in order to peel off the tumor. (E) After removing the tumor, the injured muscularis propria could be seen through the bottom of the wound. Hot biopsy forceps were used to coagulate and stop the bleeding. (F) The wound surface was sealed with titanium clips. (G) The size of the excised tumor was about 15 × 15 mm. (H) The postoperative pathological diagnosis was GST.

During ESE using a circumferential incision approach (Figure 3), the patients were placed in the left decubitus position and received intravenous anesthesia. Vital signs were monitored using a cardiac monitor. A transparent cap was placed at the front end of the endoscope.

The basic steps of this procedure were as follows: First, to mark the GST boundary, a mucosal incision knife or electric snare was used to make electrocoagulation markings approximately 5 mm outside the GST. Multipoint submucosal injections were then administered at the marked point. Next, during GST edge excision, a mucosal knife was used to open the mucosal and submucosal layers along with the marked points at the edges of the GST. A mucosal knife was then used to peel the entire GST along the muscularis propria mucosa during GST peeling. Finally, hot biopsy forceps were used for wound care to treat bleeding and expose blood vessels, and titanium clips and/or nylon rings were used to seal the wounds if necessary. The endoscope was removed after confirming that there was no active bleeding in the wound.

During the above procedure, a series of precautions are necessary, including the following (a): when marking the tumor boundary, it was not necessary to expand the area in the same way as during early cancer treatment; (b) excision of the GST should be performed at the marked boundary to avoid excessive tissue loss with subsequent bleeding and perforation; (c) the tumor capsule should be kept intact during the excision, which could be achieved by removing part of the muscularis propria; (d) the thermal coagulation of the wound should involve the soft coagulation mode as much as possible, with a minimum coagulation time to avoid postoperative electrocoagulation syndrome; (e) after the treatment was completed, the residual bloody fluid in the gastric cavity should be rinsed out to facilitate further observation; and (f) an indwelling tube should be left in the stomach postoperatively and connected to the negative pressure chamber to observe any active bleeding. Negative suction can also decrease the pressure and tension in the stomach, thereby reducing the risk of delayed perforation.

#### 2.4.2 ESE with a surface incision

**FIGURE 4:**
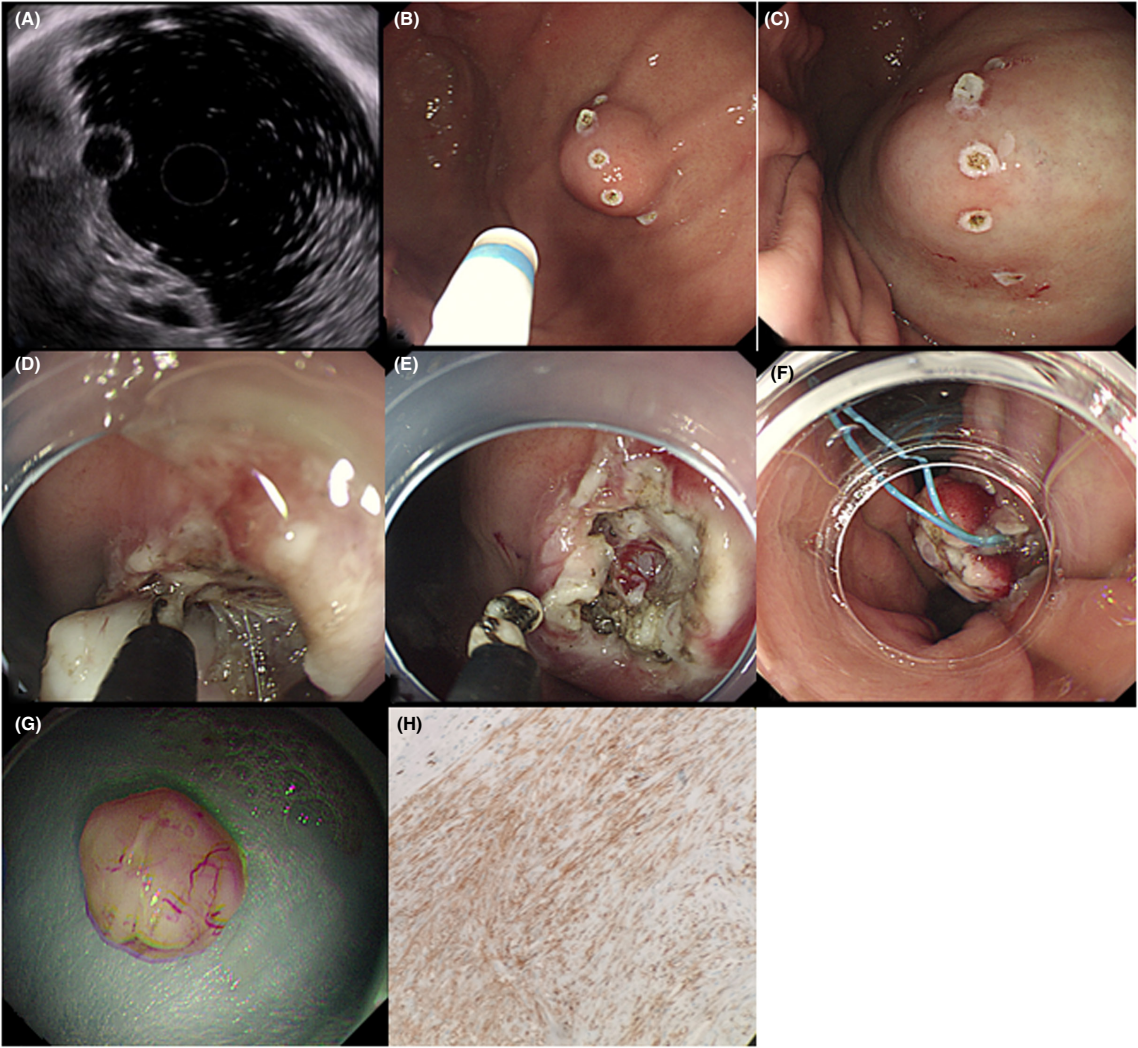
Endoscopic submucosal excavation surface incision approach. (A) Gastric stromal tumor (GST) located in the frontal wall of the gastric fundus. EUS showed that the tumor was partially an exophytic growth and originated from the muscularis propria. (B) A mucosal knife was used to create electrocoagulation markings around the GST surface. (C) Submucosal injections were performed surrounding the GST. (D) A mucosal knife was used to make a “C”‐shaped incision to open the mucosa and submucosa layers and expose the white tumor body. (E) Image showing that the GST was connected to the muscularis propria. The IT‐Knife2 was used to remove part of the muscularis propria at the bottom of the tumor. (F) After excising the tumor, the injured muscularis propria can be seen at the bottom of the wound. Hot coagulation was applied to stop the bleeding. (G) The wound could be completely sealed by nylon ring ligation. (H) The size of the removed tumor was about 12 × 14 mm. The postoperative pathological diagnosis was GST.

During ESE with a surface incision (Figure 4), the patients were placed in the left decubitus position and received intravenous anesthesia. Vital signs were monitored using a cardiac monitor. A transparent cap was placed at the front end of the endoscope.

The basic steps of this procedure were as follows. First, to mark the surface of the GST, a mucosal incision knife or an electric snare was used to make electrocoagulation markings around the GST. Subsequently, multipoint submucosal injections were performed surrounding the GST. Next, a mucosal knife cut the mucosa and submucosa layers at the top of the GST to expose the tumor surface. A mucosal knife was then used to peel and excise the tumor on both sides and the bottom of the GST during peeling and excavation. Additional submucosal injections are required to reduce the risk of perforation. Part of the muscularis propria may also be removed to ensure the integrity of the tumor when there is a close connection between the GST and the muscularis propria. Finally, the exposed blood vessels and bleeding wounds were treated using hot biopsy forceps, titanium clips, and/or nylon rings to ensure wound closure. Hemostasis was achieved before endoscope withdrawal.

During the above procedure, a series of precautions are necessary, including the following: (a) both ends of the markings should be at the edge of the GST to avoid excessively long markings; (b) the mucosal and submucosal layers should be cut precisely at the marked points on the surface of the tumor, and the surface should be incised step‐by‐step to avoid opening the GST; and (c) when excavating the tumor, the incision knife should press the muscularis propria to avoid damaging the tumor. Notably, the remaining precautions were the same as those listed for the ESE circumferential incision approach.

#### 2.4.3 ECLR

**FIGURE 5:**
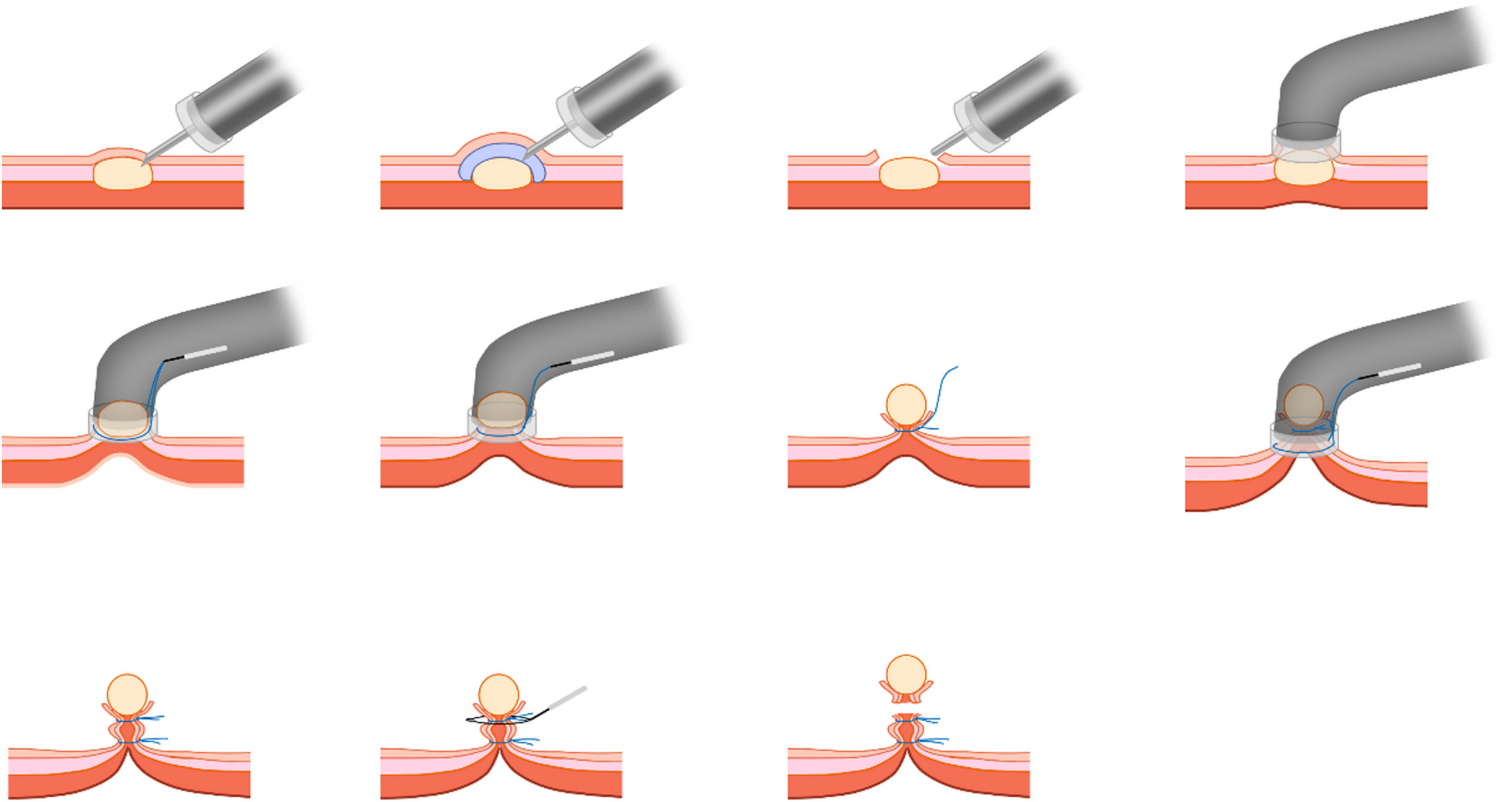
Schematic diagram of endoscopic “calabash” ligation and resection to treat endophytic gastric stromal tumor (GST). Electrocoagulation markings were made on the tumor surface, submucosal injection was performed, a transparent ligation cap was applied, and the tumor was exposed after opening the mucosa and submucosa layers. Ligation with two nylon rings was performed to form a “calabash,” the GST in the upper part of “calabash” was removed by the snare, and ligation of the lower part was completed to avoid perforation.

**FIGURE 6:**
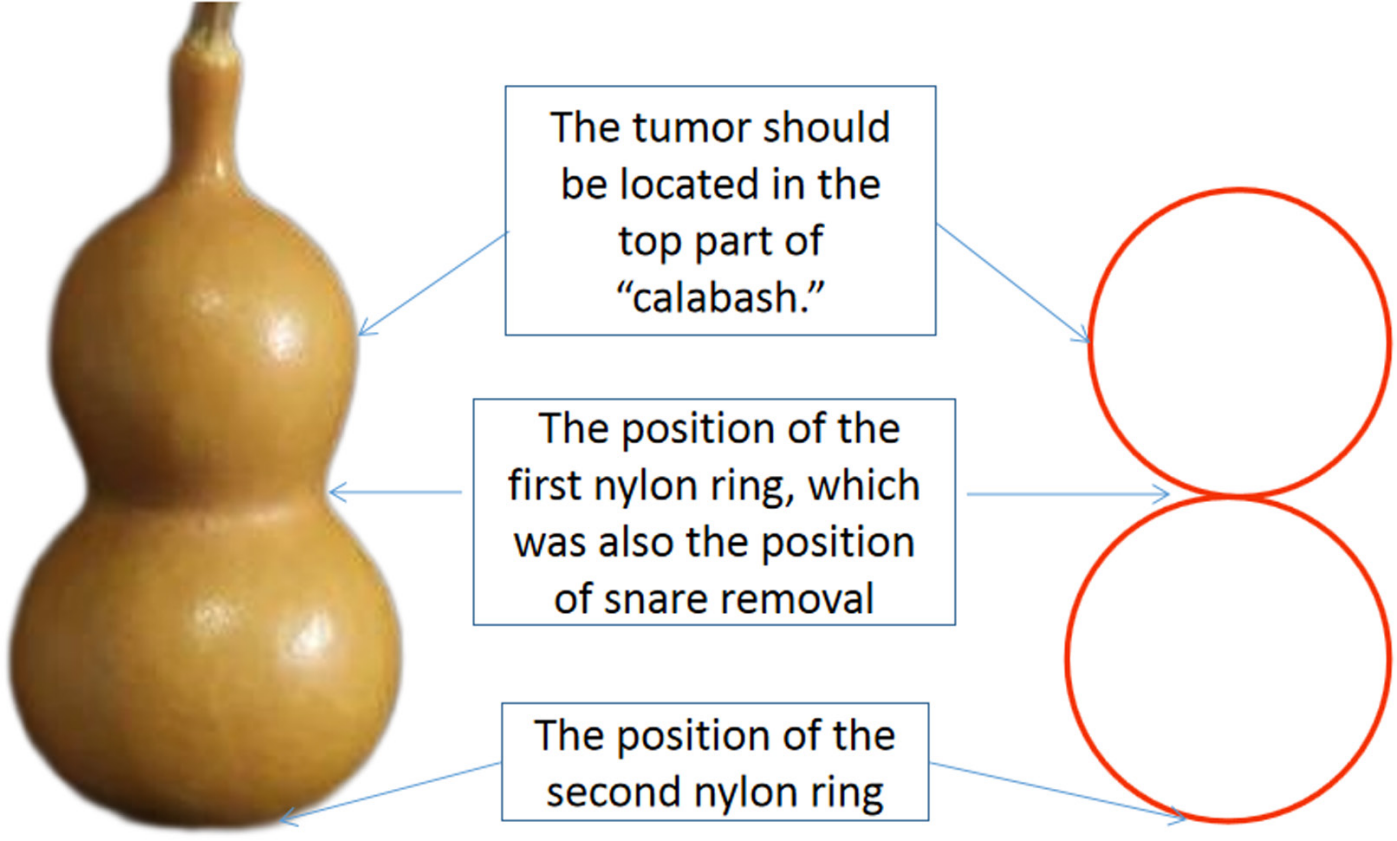
Schematic diagram of endoscopic “calabash” ligation and resection to show the positions of the two nylon rings. The first nylon ring was placed at the bottom of the tumor, and the second nylon ring was placed to tighten the full‐thickness stomach wall to form a “calabash.” An electric snare was used to excise the upper part of the “calabash” containing the tumor. The lower part of the “calabash” was fully tightened by the nylon ring to avoid perforation.

**FIGURE 7:**
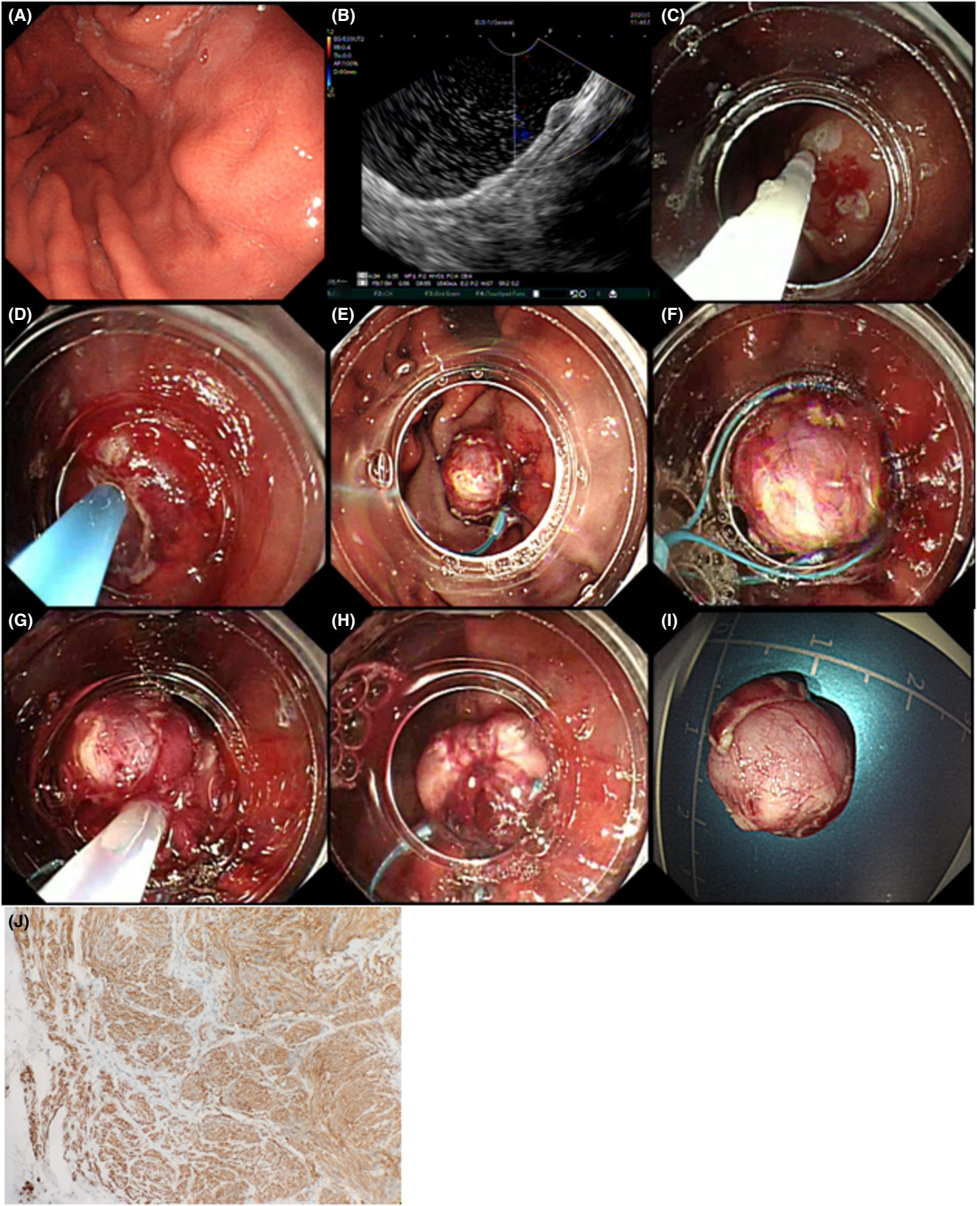
A gastric stromal tumor (GST) treated by endoscopic “calabash” ligation and resection. (A) The GST was located in the gastric fundus, close to the gastric body. (B) The tumor originated from the muscularis propria as seen under endoscopic examination. (C) Under the “U” inversion of the endoscope, submucosal injection was performed after marking the GST surface by electrocoagulation. (D) A transparent cap was placed at the tip of the endoscope. A mucosal knife was used to open the mucosa and submucosa layers and expose the tumor. (E) Under negative suction, the nylon ring was tightened at the bottom of the tumor. (F) Under further negative suction, the second nylon ring was placed to form a “calabash.” (G) Snare excision was performed to remove the upper part of “calabash.” (H) The bottom nylon ring was tightened to avoid perforation. Further ligation can be reinforced to avoid delayed wound perforation. (I) The excised tumor measured about 15 × 15 mm. (J) The postoperative pathological diagnosis was GST.

**FIGURE 8:**
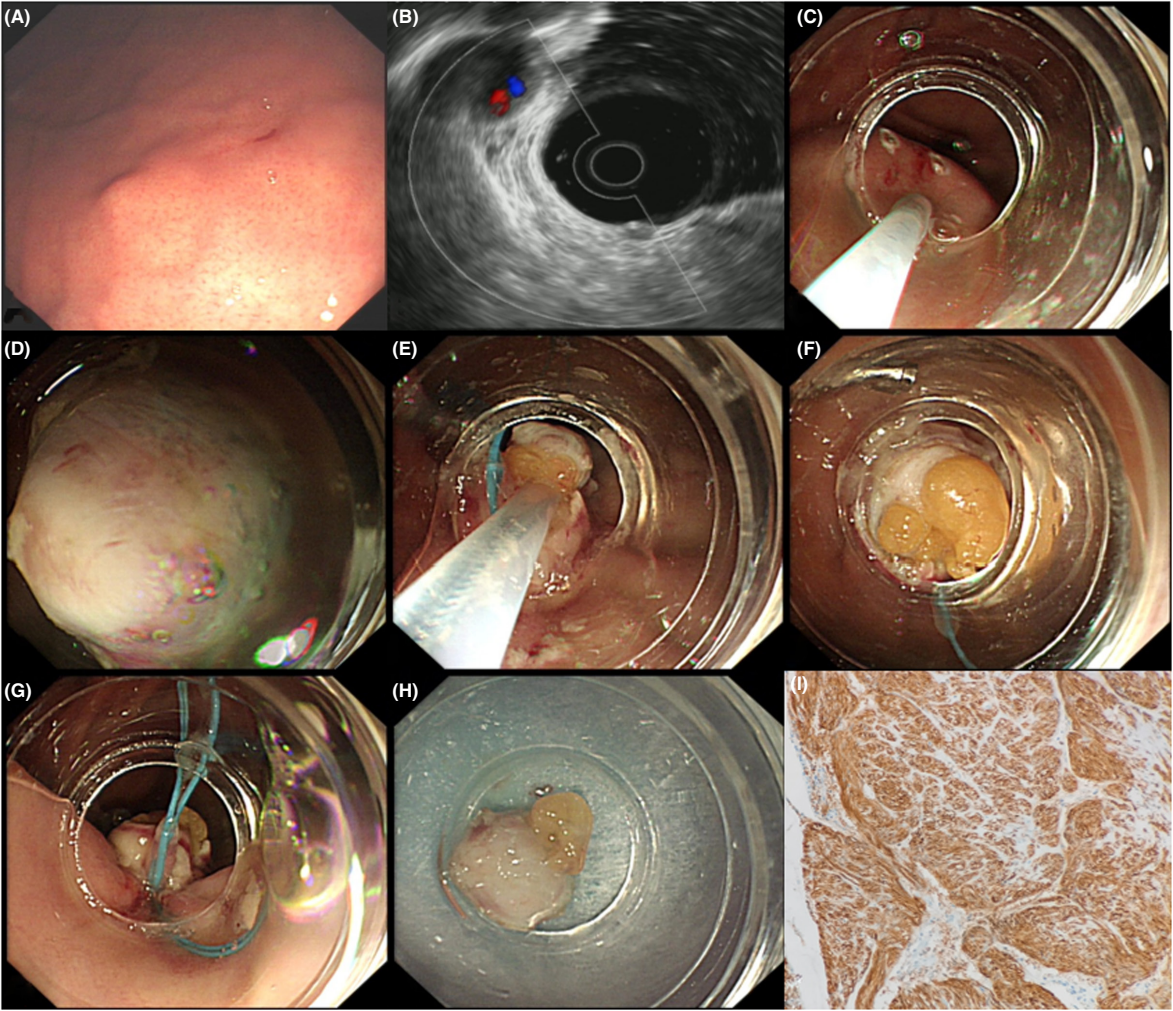
Endoscopic “calabash” ligation and resection full‐thickness ligation to resect a gastric stromal tumor (GST). (A) The GST was visible at the gastric fundus. (B) The tumor originated from the muscularis propria according to the endoscopic ultrasound evaluation. (C) A submucosal injection was performed after marking the GST surface by electrocoagulation. (D) A transparent cap was placed at the tip of the endoscope. A mucosal knife was used to open the mucosa and submucosa layers and expose the tumor. (E) Two nylon rings were applied to perform full‐thickness ligation to form a “calabash.” Then, snare excision was used to remove the upper part of the “calabash.” (F and G) After removing the upper part of the “calabash” above the first nylon ring, fat tissue in the stomach wall was exposed. The full‐thickness stomach wall in the lower part of the “calabash” was tightly ligated by the second nylon ring to avoid perforation. (H) The excised tumor measured 6 × 8 mm. (I) the postoperative pathological diagnosis was GST.

**FIGURE 9:**
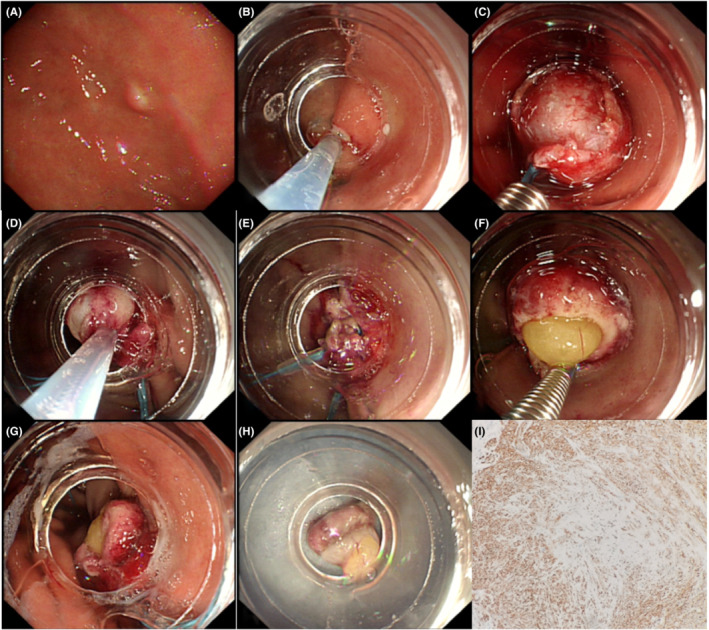
Stomach perforation during endoscopic “calabash” ligation and resection treatment of a gastric stromal tumor (GST). (A) The GST was located in the gastric fundus. (B) Submucosal injection was performed after marking the GST surface by electrocoagulation. (C) A transparent cap was placed at the tip of endoscope. A mucosal knife was used to open the mucosa and submucosa layers and expose the tumor. Then, nylon rings were applied to form a “calabash.” (D) An electric snare was used to remove the tumor. (E) The local resection site was too deep and a small perforation was visible. (F) Under negative‐pressure suction, a nylon ring was applied to establish full‐thickness ligation of the stomach wall. The fat tissue outside the stomach wall was sucked into the stomach lumen. (G) Ligation completely closed the perforation. (H) The excised tumor measured 6 × 10 mm. (I) The postoperative pathological diagnosis was GST.

During ECLR (Figures 5, 6, 7, 8, 9), the patients were placed in the left decubitus position and administered intravenous anesthesia. Vital signs were monitored using a cardiac monitor. A transparent cap was then prepared.

The basic steps of this procedure were as follows. First, to mark the surface of the tumor, a mucosal incision knife or an electric snare was used to make one straight or cross electrocoagulation marking on the surface of the tumor. Multipoint submucosal injections were then administered at the edge of the tumor. Next, a mucosal incision knife or an electric snare was used to make a straight or cross incision in the mucosal and submucosal layers at the top of the GST to expose the tumor. Then, during “calabash” ligation with a nylon ring, the tumor was sucked into the transparent ligation cap at the tip of the endoscope. At this stage, the first nylon ring was placed at the bottom of the tumor, whereas the second nylon ring was placed under the first nylon ring after suction was applied again to suck the tumor into the transparent ligation cap. These two nylon rings and the tumor looked like a “calabash,” with the GST at the top of the “calabash.” During tumor resection, an electric snare was used to cut the “calabash” above the first nylon ring to remove the top half of the “calabash.” The bottom half of the “calabash” was left intact to avoid perforation or bleeding. Finally, a nylon ring and/or titanium clip was used to ligate or clamp the wound.

During the above procedure, a series of precautions are necessary, including the following: (a) the one straight or cross incision should cover the long diameter of the tumor to allow the tumor to be sucked out completely; (b) suction should be applied after the mucosal and submucosa layers of the tumor surface are cut open, and further dissection might be required if the tumor is not able to be sucked out; (c) the suction should be applied to its maximum negative pressure (to form an entirely white tumor body under the microscope) to suck the entire tumor into the ligation cap, while the assistant slowly tightens the nylon ring; (d) the GST usually has a higher density than the muscularis propria; however, even if part of the tumor does not entirely enter the ligation cap, the nylon ring should slide to the bottom of the tumor and tighten at the muscularis propria layer; (e) the second nylon ring must be positioned to tighten the full‐thickness of the stomach wall, with the excision made above the first nylon ring to avoid perforation; (f) in cases of perforation, the nylon ring could be used again to perform full‐thickness ligation followed by sealing with a titanium clip; and (g) during negative‐pressure suction, the stomach wall protrudes into the gastric lumen; in this case, the nylon ring can ligate the full‐thickness of the stomach wall. In addition, when bleeding occurs from the serosal layer, it can be sucked into the gastric lumen to stop the bleeding.

### 2.5 Evaluations of complications

**FIGURE 10:**
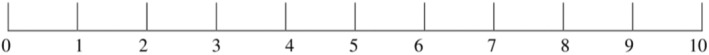
Numerical pain score chart. A scale with numbers ranging from 0–10 was used to mark different pain intensities. A score of 0 indicates no pain, 10 indicates the most severe pain, scores ≤4 indicates mild pain (the pain does not affect the individual's sleep), scores of 4–6 indicate moderate pain, and scores ≥7 indicate severe pain (pain causing an inability to sleep or causing the individual to wake up from sleep).

Potential complications include the following: (a) resection failure, defined by an inability to remove the GST with endoscopy or if the procedure is switched to a surgical operation due to significant complications; (b) intraoperative bleeding, defined by bleeding >100 ml or bleeding that was difficult to control under endoscopy; (c) intraoperative perforation, defined as a full‐thickness defect of the stomach during the procedure; (d) delayed postoperative bleeding, referring either to a postoperative bright red discharge in the gastric drainage tube or hematemesis or active bleeding or thrombosis formation revealed during endoscopic re‐examination; (f) delayed postoperative perforation, defined by a full‐thickness defect found in the stomach after the procedure; (g) intraperitoneal infection, defined by the presence of fever, an elevated white blood cell count, increased neutrophil percentage, high procalcitonin and C‐reactive protein levels, and/or CT examination revealing ascites and/or abscess formation; (h) other postoperative infections, referring to infections in other body parts except the abdominal cavity, such as the respiratory tract or chest; (i) postoperative electrocoagulation syndrome, characterized by fever, local abdominal tenderness, rebound tenderness, or elevated white blood cell count (≥10.8 × 10^9^/L) within 2 days after the endoscopic procedure, with stomach perforation ruled out based on the abdominal X‐ray or CT examination; and (j) postoperative abdominal pain, where the pain intensity is graded according to a pain score chart if there is postoperative abdominal pain (Figure 10).

### 2.6 Postoperative management

After the operation, the gastric tube was connected to a negative‐pressure chamber to observe any delayed bleeding. Vital signs were monitored at the bedside. Next, proton pump inhibitors were provided together with intravenous nutritional supportive therapy to suppress gastric acid. Later, if they are stable with no complications such as bleeding, perforation, or infection, the patient can attempt oral liquid intake 24 h after the procedure. However, an extended fasting time and antibiotics may be necessary for patients with large intraoperative wounds and significant postoperative abdominal pain, bleeding, or infection. Similarly, gastrointestinal decompression and antibiotics should be administered if a large perforation occurs during the operation. After hospital discharge, oral proton pump inhibitor suppression therapy was continued for 8 weeks. Finally, imatinib mesylate treatment and oncology and surgery follow‐up are required in patients with postoperative pathology showing intermediate‐ or high‐risk tumors.^15^

### 2.7 Discharge criteria

To be discharged from the hospital, patients should exhibit no bleeding, perforation, infection, or other complications; have no complaints of discomfort after eating liquid foods for 2 days; and show the ability to take care of themselves with a pain intensity score ≤2 points.

### 2.8 Follow‐up

Patients were asked to return to the outpatient clinic 1 week after hospital discharge and were instructed to resume normal eating and exercise after appropriate assessments. Telephone follow‐ups were also performed 2 weeks after hospital discharge to evaluate delayed complications, such as bleeding, perforation, or infection. All patients were recommended to undergo repeat endoscopic examinations at 3, 6, and 12 months after hospital discharge to assess wound healing and tumor recurrence. Low‐risk patients could undergo CT examinations every 6–12 months within 5 years after discharge, while high‐risk patients were recommended to undergo contrast‐enhanced CT examinations every 3–6 months in the first 3 years after discharge, and then twice a year after discharge.^16^ During follow‐up visits, the endoscopic procedure or surgical treatment was repeated if residual or recurrent tumors were found.

### 2.9 Statistical analysis

All statistical analyses were performed using SPSS version 25.0 (IBM Corporation, Armonk, NY, USA). Propensity score matching (PSM) was used to avoid retrospective biases, caliper was set as 0.02. Age, sex, complete endoscopic resection rates, endoscopic procedure durations, tumor lengths, treatment complications (bleeding, perforation, infection, and abdominal pain), lengths of hospital stays, medical expenses, and tumor recurrence rates were compared between the study and control groups. Continuous data were presented as mean ± standard deviation and were compared using the independent‐samples *t*‐test. Categorical data were presented as percentages (%) and were compared using the chi‐square test. Non‐normal distributions or non‐uniform variances were compared using the non‐parametric Wilcoxon test. *p <* 0.05 was considered to be statistically significant.

## 3 RESULTS

### 3.1 Patient enrolment

**FIGURE 11:**
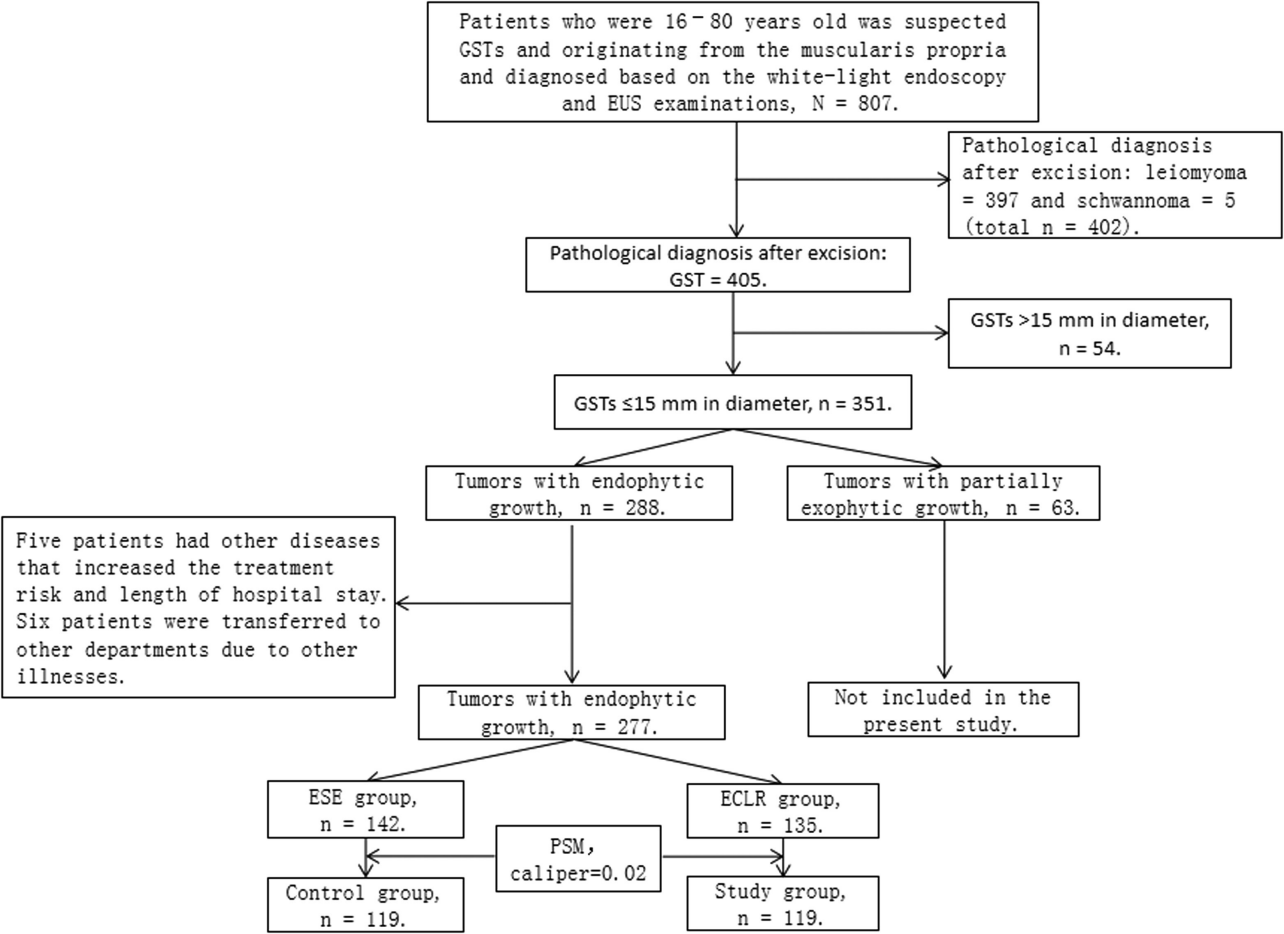
Participant selection flowchart

The patient enrolment process is shown in Figure 11. From February 2019 to December 2020, 807 patients were suspected of having GSTs after white‐light endoscopy and EUS examinations. After obtaining informed consent, they underwent endoscopic resection and biopsy. Pathological reports showed 397 cases of leiomyoma and five cases of schwannoma. A total of 405 patients were confirmed to have GSTs. Among them, 54 patients had GSTs >15 mm in diameter and 351 patients had GSTs ≤15 mm in diameter according to their preoperative EUS results. Of those patients with GSTs ≤15 mm in diameter, 288 had endophytic growth and 63 had partial exophytic growth, according to their EUS examinations. No GST showed complete exophytic growth. A total of 147 patients with endophytic GSTs originating from the muscularis propria and with a diameter of ≤15 mm underwent ESE. Two patients with other diseases had increased treatment risks and lengths of hospital stays. Three patients were transferred to different departments due to other illnesses. In total, there were 142 patients included in the control group. Additionally, 141 patients with endophytic GSTs ≤15 mm in diameter originating from the muscularis propria underwent ECLR. Three patients with other diseases had increased treatment risks and lengths of hospital stays. Three patients were transferred to different departments due to other illnesses. Thus, there were 135 patients included in the study group in total. After propensity score matching (PSM) in sex, age, GST location, and GST diameter, 119 cases in each group were included in the final analysis.

### 3.2 Comparison between the study and control groups

#### 3.2.1 Comparisons of baseline characteristics

The control group included 74 women and 45 men, with a mean age of 56.92 ± 9.92 years (range, 31–79 years). There were 97 cases of GSTs ≤10 mm in diameter and 22 cases of GSTs 11–15 mm in diameter. The mean GST diameter was 7.73 ± 3.24 mm (range, 4–15 mm). GSTs were mainly located in the gastric fundus in 102 patients and in the gastric body in 17 patients. Most GSTs (118 cases) were very low risk. Only one case was classified as intermediate risk. No high‐risk cases were identified. The study group included 75 women and 44 men, with a mean age of 56.61 ± 9.45 years (range, 29–78 years). There were 104 cases of GSTs ≤10 mm in diameter and 15 cases of GSTs of 11–15 mm in diameter. The mean GST diameter was 7.64 ± 2.42 mm (range, 4–14 mm). GSTs were located in the gastric fundus in 101 cases, the gastric body in 17 cases, and the antrum in one case. All the patients had low‐risk GSTs. The detailed information is presented in Table 1.

**TABLE 1:** Comparisons of baseline characteristics between the two groups

Baseline characteristics	Control group (*n* = 119)	Study group (*n* = 119)	*p*
Sex, female/male	74/45	75/44	0.893
Age, years, mean ± SD	56.92 ± 9.92	56.61 ± 9.45	0.805
GST, *n*			
Diameter ≤ 10 mm	97	104	0.210
Diameter 11–15 mm	22	15	
GST diameter, mm, mean ± SD	7.73 ± 3.24	7.64 ± 2.42	0.803
GST location, *n*			
Fundus	102	101	0.855
Body	17	17	>0.999
Antrum	0	1	>0.999
GST cancer risk classification, n			
Very low	118	119	>0.999
Low	0	0	>0.999
Moderate	1	0	>0.999
High	0	0	>0.999

Abbreviations: GST, gastric stromal tumor; SD, standard deviation.

There were no statistically significant differences in sex (*p* = 0.893), age (*p* = 0.805), GST size distribution (*p =* 0.210), GST mean diameter (*p* = 0.803), GST location (*p* = 0.855, >0.999, and >0.999 in the gastric fundus, body, and antrum, respectively), or GST risk classification (*p* > 0.999) between the two groups (Table 1).

#### 3.2.2 Comparison of endoscopic outcomes

Postoperative pathological biopsy revealed intact GST capsules in all the cases. All the GSTs were completely resected (Table 2).

**TABLE 2:** Comparisons of resection outcomes between the two groups

Resection, *n* (%)	Control group (*n* = 119)	Study group (*n* = 119)	*p*
Complete resection of intact tumor	119 (100%)	119 (100%)	>0.999
Complete resection	0	0	>0.999
Incomplete resection	0	0	>0.999

#### 3.2.3 Comparisons of intraoperative complications

There were no cases of intraoperative hemorrhage in either the study or the control group. There was also no instance of a failed endoscopic case that required the patient to be switched to surgery in either group. The incidence of intraoperative perforation was 9.24% (11/119) and 2.52% (3/119) in the control and study groups, respectively. The incidence of intraoperative perforation was lower in the study group than in the control group (*p =* 0.029) (Table 3).

**TABLE 3:** Comparisons of intraoperative complications between the two groups

Intraoperative complications, *n* (%)	Control group (*n* = 119)	Study group (*n* = 119)	*p*
Hemorrhage	0	0	>0.999
Switched to surgical operation due to failed endoscopic treatment	0	0	>0.999
Perforation	11 (9.24%)	3 (2.52%)	0.029

#### 3.2.4 Comparisons of postoperative complications

The scores of postoperative abdominal pain were 2.16 ± 1.37 points (range, 0–7 points) and 1.55 ± 1.0 points (range, 0–5 points) in the control and study groups, respectively, and the study group's pain score was significantly lower than the control group's (*p <* 0.001). One patient in the control group had delayed postoperative bleeding and required two units of red blood cells. Postoperative delayed bleeding was not observed in the study group. There was no statistically significant difference in postoperative delayed bleeding between the two groups (*p* > 0.999). There were two cases (incidence, 1.68%) of postoperative delayed perforation in the control group and no cases of postoperative delayed perforation in the study group. There was no significant difference in postoperative delayed perforation between the two groups (*p =* 0.478). Six patients in the control group (incidence, 5.04%) had postoperative intraperitoneal infections, whereas the study group had no cases of postoperative intraperitoneal infection. The incidence of postoperative intraperitoneal infection in the study group was lower than that in the control group (*p =* 0.039). There were two cases and one case of postoperative respiratory infections in the control and study groups, respectively (the incidences were 1.68% and 0.84%, respectively). There was no significant difference in postoperative respiratory infections between the two groups (*p* > 0.999). The incidence of postoperative coagulation syndrome was 0.00% (0/119) in the study group, which was lower than that of 5.88% (7/119) in the control group (*p =* 0.021) (Table 4).

**TABLE 4:** Comparisons of postoperative complications between the two groups

Postoperative complications	Control group (*n* = 119)	Study group (*n* = 119)	*p*
Abdominal pain score, points, mean ± SD	2.16 ± 1.37	1.55 ± 1.00	<0.001
Delayed hemorrhage, *n* (%)	1 (0.84%)	0 (0.00%)	>0.999
Units of red blood cell transfusion, *n*	2	0	
Delayed perforation, *n* (%)	2 (1.68%)	0	0.478
Delayed intraperitoneal infection, *n* (%)	6 (5.04%)	0	0.039
Respiratory tract infection, *n* (%)	2 (1.68%)	1 (0.84%)	>0.999
Electrocoagulation syndrome, n (%)	7 (5.88%)	0 (0.00%)	0.021

Abbreviation: SD, standard deviation.

#### 3.2.5 Comparison of endoscopic procedure durations

The durations of the endoscopic procedures in the control and study groups were 39.7 ± 10.45 min (range, 25–85 min) and 22.0 ± 5.74 min (range, 12–42 min), respectively, and the study group's duration was significantly shorter than the control group's (*p <* 0.001) (Table 5).

**TABLE 5:** Comparisons of treatment outcomes between the two groups

Treatment outcomes	Control group (*n* = 119)	Study group (*n* = 119)	*p*
Duration of procedure, min	41.0 ± 11.7	22.0 ± 5.7	<0.001
Length of hospital stay, days	6.5 ± 3.9	5.1 ± 1.6	0.001
Medical expenses, Chinese yuan	18,060.3 ± 8675.1	13,570.4 ± 2920.4	<0.001

*Note*: All data are presented as mean ± standard deviation values.

#### 3.2.6 Comparison of lengths of hospital stay

The lengths of the hospital stays were 6.35 ± 4.01 days (range, 2–31 days) and 5.07 ± 1.64 days (range, 2–13 days) in the control and study groups, respectively, and the study group's hospital stays were significantly shorter than the control group's (*p* = 0.001) (Table 5).

#### 3.2.7 Comparison of medical expenses

The medical expenses were 17,502.87 ± 7514.01 Chinese yuan (range, 9707–77,451 Chinese yuan) and 13,686.84 ± 3016.65 Chinese yuan (range, 7735–24,086 Chinese yuan) in the control and study groups, respectively, and the study group's expenses were significantly lower than the control group's (*p <* 0.001) (Table 5).

#### 3.2.8 Comparisons of follow‐up outcomes

There were no statistically significant differences in the follow‐up durations between the two groups (Table 6). Neither group experienced tumor recurrence, metastasis, or death during the follow‐up period.

**TABLE 6:** Comparisons of follow‐up outcomes between the two groups

Follow‐up outcomes	Control group (*n* = 119)	Study group (*n* = 119)	*p*
Follow‐up duration, month,			
Mean ± standard deviation	14.39 ± 4.89	14.39 ± 6.40	0.992
Median (range)	13 (6–25)	13 (6–25)	
Tumor recurrence, *n*	0	0	>0.999
Metastasis, *n*	0	0	>0.999
Mortality, *n*	0	0	>0.999

## 4 DISCUSSION

A GST is a common type of GSMT that carries a risk of malignant transformation. The findings on white‐light endoscopic and EUS images are similar between sGSTs and leiomyomas; thus, pathological immunohistochemical examinations should be used to differentiate them.^17^ However, GSTs with a thick fibrous capsule may not be good candidates for EUS‐FNA puncture, which could result in insufficient biopsy for an accurate histopathologic diagnosis.^18^ The diagnostic accuracy of EUS‐FNA has been reported to be 71% for sGSTs <2 cm in diameter.^19^ In addition, tissue obtained through endoscopic biopsy or EUS‐FNA cannot be used for risk assessment.^20^ Therefore, the accurate diagnosis of GST and its potential malignant transformation requires the pathological evaluation of gross specimens. The present study aimed to demonstrate the efficacy and safety of a new endoscopic technique for the treatment of GSTs ≤15 mm in diameter that originate from the muscularis propria. Our results showed that this technique was effective, relatively simple, safe, and inexpensive for treating this type of GST.

Most GSTs are located on the gastric fundus. ESE or EFTR is often required to adopt a “U”‐shaped reverse position, which complicates the operation and exposes of the bottom of the tumor. Therefore, a traction technique may be beneficial for this procedure. During ECLR, the endoscope is positioned close to the tumor on the frontal or lateral side after incising the mucosal and submucosal layers on the tumor surface. Subsequently, the tumor can then be easily sucked into the ligation cap through negative‐pressure suction without external traction. In both groups, all GSTs were entirely removed in our study, which was confirmed by postoperative pathological examinations.

No intraoperative bleeding (bleeding volume > 100 ml) was reported in the study and control groups. Adequate surgical field exposure is essential during the ESE procedure, as this protects the major vessels and prevents uncontrollable hemorrhage. During the ECLR procedure, the bottom of the tumor or even the full‐thickness stomach wall can be bandaged in a manner similar to that during surgical operations. This can lead to satisfactory hemostasis.

With the continuous advancement of endoscopic technology, the current treatment of small perforations using endoscopic procedures can result in satisfactory outcomes. Severe complications rarely occur in these patients. As it is necessary to ensure the integrity of the tumor during the ESE procedure, the tumor is excavated close to the muscularis propria, and part of the muscularis propria can be removed. This can result in frequent intraoperative perforations. Therefore, attention should be paid to avoiding hemorrhage and treatment failure due to large perforations. In the present study, the control group included 11 cases (9.24%) of intraoperative perforations, all of which were successfully sealed and treated using an endoscope. The study group had three patients (2.52%) with perforations related to an inadvertent cut made at the lower half of the “calabash” during the snare resection step. The perforations were completely closed under endoscopic view using nylon rings. The incidence of intraoperative perforation was lower in the study group than in the control group (*p =* 0.029).

The control group included one patient with delayed bleeding who received two red blood cell transfusion units. Repeat endoscopic examination revealed delayed bleeding due to early dislodgement of the titanium clip, which ceased after the endoscopic treatment. No statistically significant difference was observed in the rate of delayed postoperative perforation between the control and study groups. However, the control group had two cases (1.68%) with delayed postoperative perforations, and the study group had no cases of postoperative perforation. Emergency endoscopy revealed that both perforations in the control group were caused by early dislodgement of the titanium clips. Under an endoscope, the titanium clip and nylon ring were applied to create “purse‐string” sutures to seal all perforations without requiring surgery. There was no delayed perforation in the study group due to firm ligation of the wound with the nylon ring. To avoid early dislodgement of the titanium clip, close attention should be paid to avoid overlapping the clips (e.g., one clip placed on top of another). Postoperative gastric decompression using an indwelling gastric tube could reduce stomach tension and minimize the risk of early dislodgement of titanium clips.

In the control group, there were six cases (5.04%) of postoperative intraperitoneal infection, two of which were due to delayed perforation. The other four cases were considered to be related to the leakage of lavage fluid into the abdominal cavity during the procedure. Therefore, once perforation is suspected during the process, excessive lavage should be avoided to minimize the risk of secondary intraperitoneal infections. After antibiotic treatment, all patients recovered completely without surgical intervention. There were no reports of postoperative abdominal infection in the study group. The incidence of postoperative abdominal infection in the study group was lower than that in the control group (*p =* 0.039).

One patient (0.84%) in the study group and two patients (1.68%) in the control group developed postoperative respiratory infections. These were all considered to be related to aspiration during the endoscopic procedure, and all patients recovered following antibiotic treatment. There was no significant difference between the two groups (*p* > 0.999).

Seven patients (5.88%) and one patient (0.00%) developed postoperative electrocoagulation syndrome in the control and study groups, respectively, and the incidence was significantly higher in the control group than in the study group (*p =* 0.021). The occurrence of postoperative electrocoagulation syndrome is mainly due to the coagulation mode and duration. Therefore, efforts should be made to use the resection mode, soft coagulation type, and the minimum coagulation duration (especially with strong intensity) to decrease the risk of postoperative electrocoagulation syndrome.

The mean postoperative abdominal pain score in the study group was 1.55 (±1.0) points, which was lower than the mean pain score of 2.16 (±1.37) in the control group (*p <* 0.001). Most patients had mild abdominal pain, which did not affect their daily routines. The statistical difference in the pain scores between the two groups was not clinically significant.

The control group used the ESE treatment method, and the mean procedure and length of hospital stay durations were 39.7 ± 10.45 min and 6.35 ± 4.01 days, respectively. These results are consistent with those of previous studies.^10^, ^21^, ^22^, ^23^ The study group underwent the ECLR procedure, and the procedure and length of hospital stay durations were 22.0 ± 5.74 min and 5.07 ± 1.64 days, respectively. These values were significantly lower than those in the control group (*p* = 0.001). The medical expenses were 13,686.84 ± 3,016.65 and 17,502.87 ± 7,514.01 Chinese yuan in the study and control groups, respectively, and the study group's expenses were significantly lower than the control group's (*p <* 0.001).

In our control group, one case of sGST was found to carry an intermediate risk (gastric body 7 mm in diameter). The remaining patients were at extremely low risk. The patient with intermediate‐risk GST was transferred to the oncology department for imatinib‐targeted therapy, and close follow‐up visits involving endoscopy and CT examinations were arranged. During the 16‐month follow‐up period, there was no recurrence or metastasis of the GST. A previous study found that GSTs in the gastric body are more likely to be aggressive.^3^ Therefore, more attention should be paid to the follow‐up and monitoring of patients with GST lesions in the gastric body.

This study has several limitations. First, this was a single‐center retrospective study with a relatively small number of patients and short follow‐up time. Biases can be occurred by the small sample size. Different endoscopes and physicians with diverse technical skills may have affected the study results. Future prospective, multicenter, randomized clinical trials with large sample sizes are needed to confirm the study results.

## 5 CONCLUSIONS AND RECOMMENDATIONS

In the present study, we used either ESE (control group) or ECLR (study group) to treat endophytic sGSTs ≤15 mm in diameter that originated from the muscularis propria. All patients achieved complete resection (100%), fewer intraoperative and postoperative complications, and the study group had a shorter procedure duration and length of hospital stay, and lower medical expenses. Therefore, ECLR can be recommended as a new endoscopic procedure to treat this type of sGST.

## AUTHOR CONTRIBUTIONS

**minsi peng:** Formal analysis (equal); writing – original draft (equal). **Haotian Zeng:** Writing – original draft (equal). **Zhuliang Zhang:** Formal analysis (equal). **Zeming Chen:** Investigation (equal); validation (equal). **Ting Long:** Investigation (equal); validation (equal). **Lisheng Wang:** Conceptualization (equal); writing – review and editing (equal). **zhenglei Xu:** Conceptualization (lead); data curation (lead); writing – review and editing (equal).

## CONFLICT OF INTEREST

The authors affirm that this study has no potential conflicts of interest.

## FUNDING INFORMATION

Supported by Clinical research and Cultivation project of Shenzhen People’s Hospital, No.SYLCYJ202116.

## ETHICS APPROVAL STATEMENT AND PATIENT CONSENT STATEMENT

The study protocols were approved by the institutional review board of Shenzhen People’s Hospital.

## CLINICAL TRIAL REGISTRATION

China Clinical Registration and Trial Center No. ChiCTR2100042784.

## Data Availability

The original data can be reasonably obtained from the corresponding author
